# Angiotensin-I-converting enzyme inhibitory peptides from eel (*Anguilla japonica*) bone collagen: preparation, identification, molecular docking, and protective function on HUVECs

**DOI:** 10.3389/fnut.2024.1462656

**Published:** 2024-12-05

**Authors:** Huan Xiang, Hui Huang, Yanqiu Shao, Shuxian Hao, Laihao Li, Ya Wei, Shengjun Chen, Yongqiang Zhao

**Affiliations:** ^1^Key Laboratory of Aquatic Product Processing, Ministry of Agriculture and Rural Affairs, National R&D Center for Aquatic Product Processing, South China Sea Fisheries Research Institute, Chinese Academy of Fishery Sciences, Guangzhou, China; ^2^Co-Innovation Center of Jiangsu Marine Bio-industry Technology, Jiangsu Ocean University, Lianyungang, China; ^3^Collaborative Innovation Center of Seafood Deep Processing, Dalian Polytechnic University, Dalian, China

**Keywords:** eel bone collagen, ACE inhibitory peptides, peptidomics, molecular docking, HUVECs

## Abstract

**Introduction:**

Hypertension is a chronic cardiovascular disease, which can trigger some disease such as heart failure, loss of vision or kidney. There were various peptides derived from food that are recognized for their ability to inhibit ACE activity, potentially leading to a reduction in blood pressure levels *in vivo*. The primary objective of this research is to discover ACE inhibitory peptides from protein hydrolysates of eel bone collagen (EBCHs).

**Methods:**

To begin, EBCHs were created and then divided through the process of ultrafiltration. The second step involved screening of peptides capable of inhibiting ACE by combining peptidomics and molecular docking. And the mechanism by which ACE interacts with peptides has been studied. Finally, the hypotensive mechanism of identified peptide through cell experiments with HUVEC (Human Umbilical Vein Endothelial Cells).

**Results:**

Eel (*Anguilla japonica*) bone collagen was hydrolyzed by alcalase and the hydrolysate was separated into three fractions, among which the F2 displayed a higher level of ACE inhibitory activity. According to molecular docking calculations, a total of 615 peptides were identified through nano-HPLC-MS/MS, with the prediction of seven newly discovered ACE inhibitory peptides (PMGPR, GPMGPR, GPAGPR, GPPGPPGL, GGPGPSGPR, GPIGPPGPR, GPSGAPGPR). Notably, GPPGPPGL had the lowest IC_50_ value of 535.84 μM among the identified peptides, indicating its potency as an ACE inhibitor. The ACE S2 pocket formed hydrogen and hydrophobic interactions with GPPGPPGL. Lineweaver-Burk plots revealed that GPPGPPGL competitively bound to ACE’s active site residues. Treatment with GPPGPPGL significantly increased nitric oxide secretion (*p* < 0.01) and decreased endothelin-1 (ET-1) production in HUVECs.

**Discussion:**

Our findings suggest that combining peptidomics with molecular docking is effective for rapidly screening ACE inhibitory peptides. Future studies should assess the bioavailability and *in vivo* activity of the identified peptide GPPGPPGL from EBCHs.

## Graphical abstract

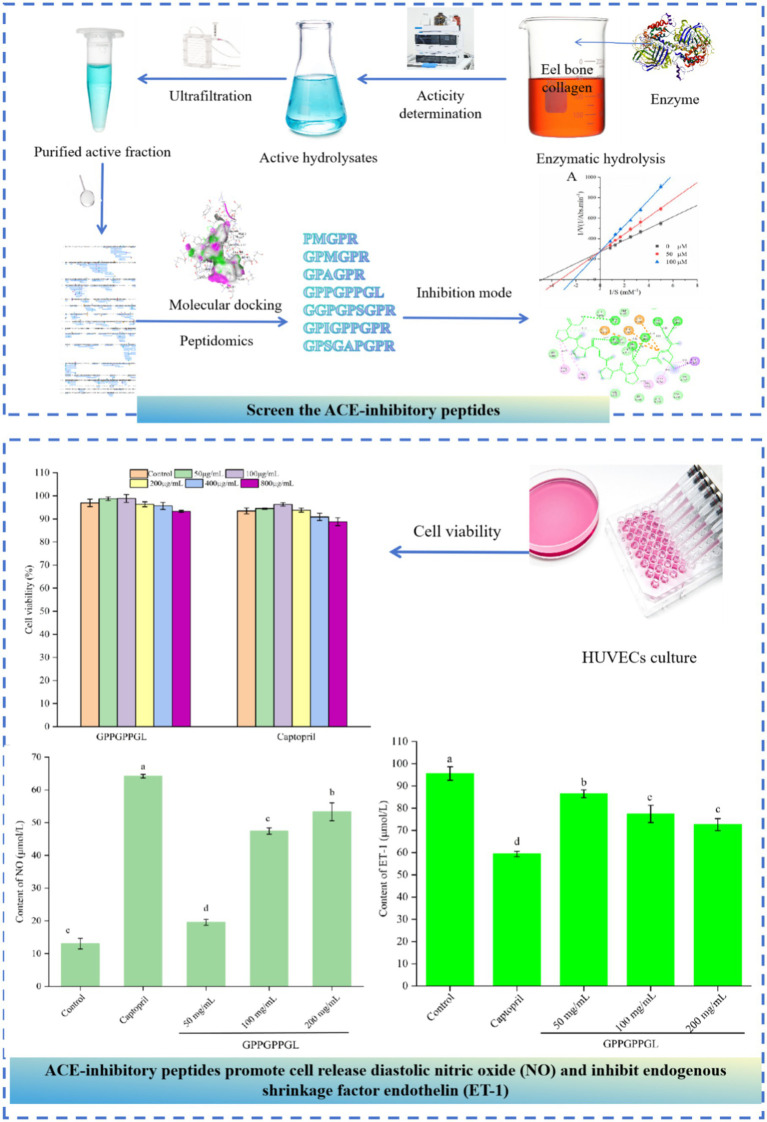

## 1 Introduction

Hypertension is a chronic cardiovascular disease that can trigger some diseases such as heart failure, loss of vision, or kidney disease (1). Effective treatment of hypertension can be achieved by focusing on angiotensin-I-converting enzyme (ACE), an enzyme that can be targeted. ACE, which contains a zinc ion in its active site, is a dipeptidyl carboxypeptidase that plays a crucial role in both the renin–angiotensin system and the kallikrein–kinin system (2). ACE transforms angiotensin I (Ang I) into Ang II or hinders the degradation of bradykinin, resulting in elevated blood pressure (3). Thus, suppressing the function of ACE proves to be a successful approach in controlling blood pressure, leading to the creation of various artificial ACE inhibitors used as medications for hypertension (4). Currently, the primary approach to managing hypertension involves the utilization of artificial medications such as benazepril, captopril, and enalapril, which can result in adverse reactions including coughing, skin irritations, and alterations in taste (5). Therefore, the identification of organic ACE inhibitors with negligible adverse reactions is exceptionally important.

There were various peptides derived from food that are recognized for their ability to inhibit ACE activity, potentially leading to a reduction in blood pressure levels *in vivo* (6). Several naturally occurring peptides that inhibit ACE have exhibited a lack of similar adverse reactions when compared to synthetic medications (7). At present, polypeptides with lowering blood pressure were prepared from different marine protein sources, such as *Gracilariopsis lemaneiformis* (Gln-Val-Glu-Tyr, IC_50_: 474.36 μM) (8), *Euphausia superba* (Ile-Pro-Ile-Lys, IC_50_: 57.4 μM) (9), and *Cyclina sinensis* (Trp-Pro-Met-Gly-Phe, IC_50_: 789 μM) (10).

*Anguilla japonica*, also known as eel, is a type of aquatic food that contains high levels of protein, fat, vitamin A, vitamin E, and other nutrients (11). It is particularly abundant in collagen. Eel processing is mainly based on grilled eel, most of which is exported abroad. During the eel processing of eels, various by-products such as fish heads and bones are generated. These by-products are mainly used in the production of animal feed or discarded. Eel bones have approximately 13.25% of protein, 19.18% of lipid, 5.63% of ash, and 56.96% of moisture content (12). Some bioactive peptides were found in eel or eel by-products, including antioxidant peptides, anti-bacterial peptides (13), and calcium-chelated peptides (14). Furthermore, a new peptide with antioxidant properties [Ile-Val-Gly-Gly-Phe-Pro-His-Tyr-Leu (1,189 Da)] was discovered in sand eel *Hypoptychus dybowskii* (15), while another peptide with anti-oxidative effects (Leu-Gly-Leu-Asn-Gly-Asp-Asp-Val-Asn, IC_50_ 78.5 μM) was found in conger eel (*Conger myriaster*) (16). A total of 11 ACE inhibitory peptides obtained from fermented milk were evaluated through the integration of peptidomics and *in silico* prediction (17). In addition, a total of 96 peptides ranging from 5 to 12 amino acids in length were chosen for peptide-ACE interaction analysis through molecular docking and simulation (18). In these studies, screening combined with the molecular docking results was demonstrated as an alternative method of identifying peptides.

The primary objective of this research was to discover ACE inhibitory peptides from protein hydrolysates of eel bone collagen (EBCHs). First, EBCHs were created and then divided through the process of ultrafiltration. The second step involved screening of peptides capable of inhibiting ACE by combining peptidomics and molecular docking. Third, the mechanism by which ACE interacts with peptides was studied. Finally, the hypotensive mechanism of the identified peptide was studied using cell experiments with HUVEC. Using the findings from this study, it is possible to rapidly and efficiently screen ACE inhibitory peptides, thereby supporting a wide range of applications.

## 2 Materials and methods

### 2.1 Materials and reagents

Eel bone was provided by Guangdong Shunde Donglong Company Grilled Eel Co., Ltd. (China). Alcalase (8,000 U/mL, protease from *Bacillus licheniformis*) and pepsin (10,000 U/mg, pig gastric mucosa) were purchased from Novozymes (Beijing, China). ACE (≥2.0 U/mg, rabbit lung) was purchased from Shanghai Yuanye Biotechnology Co., Ltd. (Shanghai, China). N-Hippuryl-His-Leu (HHL, CAS: 207386–83-2) hydrate was purchased from Nanjing Jiaoziteng Scientific Equipment Co., Ltd. (Nanjing, China). Fetal bovine serum and Dulbecco’s modified Eagle’s Medium (DMEM) were purchased from Thermo Fisher Scientific (Beijing, China). MTT Cell Proliferation and Cytotoxicity Assay Kit (Cat. No.: C0009S) was purchased from Shanghai Beyotime Biotechnology (Shanghai, China). All the other chemicals were of analytical grade.

### 2.2 Preparation of eel bone collagen hydrolysates (EBCHs)

A 5% (w/v) eel bone collagen solution was prepared. Five commercial proteases (5,000 U/g eel bone collagen) were selected to hydrolyze eel bone collagen for 4 h. Enzymatic hydrolysis temperatures and pH levels were as follows: Alcalase (50°C, pH 9.0), trypsin (37°C, pH 8.0), Protamex (45°C, pH 7.0), papain (55°C, pH 7.5), and pepsin (37°C, pH 2.0). Subsequently, EBCHs were boiled for 10 min to inactivate these proteases and centrifuged at 8000 × g for 20 min at 4°C using a GL21M refrigerated centrifuge (Changsha Xiangzhi Centrifuge Co. LTD, Hunan, China). The collected supernatant (EBCHs) was freeze-dried and stored at −18°C till use.

### 2.3 Separation of ACE inhibitory peptides

To obtain higher ACE inhibitory activity peptides, EBCH solution was passed through ultrafiltration membranes with molecular weights (MW) of 3 and 1 kDa to obtain three fractions, which were termed as F1 (MW > 3 kDa), F2 (MW 1 kDa–3 kDa), and F3 (MW < 1 kDa).

### 2.4 ACE inhibitory peptide identification through peptidomics

The EASY-nanoLC 1,200 was linked to the Q-Exactive Plus mass spectrometer (Thermo Fisher Scientific, MA, United States). The reconstitution of the samples was performed in solvent A, which consisted of 0.1% formic acid in water. Then, the peptide (4 μL) was separated using Acclaim PepMap Cl8 (75 μm × 25 cm) with a gradient that lasted for 60 min. The gradient started at 2% buffer B, which composed of 80% ACN with 0.1% formic acid, and increased gradually to 35% over 47 min. After that, it reached 100% in just 1 min and remained at that level for 12 min. The flow rate of the column was kept at 300 nL/min with a column temperature of 40°C. The device was used in a mode of data-dependent acquisition. The MS scan range (m/z) ranged from 200 to 3,000. During the collision, the resolution was set at 70 K, the automatic gain control target was 3e6, and the maximum injection time was 50 ms. The collision energy was 28 V. The resolution of MS/MS was 17.5 K, with a target automatic gain control of le5 and a maximum injection time of 45 ms. The dynamic exclusion period lasted for 30 s. Raw data were analyzed using PEAKS Studio version 10.6 (Bioinformatics Solutions Inc., Waterloo, Canada). PEAKS DB was set up to search the UniProt-*Pisum sativum* database (version 202,108, 2,357 entries), without considering any specific digestion enzyme. Oxidation and deamidation were the alterations made to the variable. The peptide was applied −10lgP ≥ 20, and the protein was applied −10lgP ≥ 0, containing at least one unique peptide.

### 2.5 Screening of ACE inhibitory peptides

Sequentially, the screening of ACE inhibitory peptides identified from F2 was performed. The *in silico* prediction of ACE inhibitory activity was assessed using the peptide ranker program, which can be found at http://distilldeep.ucd.ie/PeptideRanker/. The Peptide Ranker server utilizes a unique N-to-1 neural network to forecast the activity of bioactive peptides. Peptide Ranker was trained in the present study with a threshold of 0.8, indicating that any peptide predicted above this threshold is classified as bioactive. Furthermore, these peptides were molecular docked using AutoDock Vina. The crystal structure of ACE (entry code: 1O8A) was acquired from the Protein Data Bank. Peptide Vina scores were acquired following the docking process with 1O8A, while the binding sites were determined as spherical regions containing protein residues within a radius of 15 Å. The 3D and 2D docking images were obtained from the software Discovery Studio 2020 Client. Furthermore, molecular docking was also used to characterize interactions between peptides and ACE.

### 2.6 *In vitro* ACE inhibitory activity

The ACE inhibitory activity was determined according to Sun et al. (19) by using HPLC with slight modifications. A sample solution was mixed with borate buffer (pH 8.3, 0.1 mol/L, containing 0.3 mol/L NaCl). A volume of 50 μL of sample was mixed with 50 μL of HHL and incubated at 37°C for 5 min and then the mixture was added to ACE solution (50 μL, 0.1 U/mL) for another 30 min. Finally, HCl (100 μL, 1 mol/L) was added to the mixture to stop the reaction. The control was replaced by a Na-borate buffer solution. After the preparation of the sample, the reaction solutions were filtered through a 0.22-μm microporous membrane, and hippuric acid (HA, the reaction production) contents were determined using HPLC. The sample (10 μL) was injected on a column (C18-WP, 4.6 × 250 nm, 5 μm, 28°C), with the solvent A of 25% acetonitrile mixed 75% ultrapure water (containing 0.1% TFA) and the solvent B of ultrapure water (containing 0.1% TFA). The flow rate was set at 0.5 mL/min and measured at 228 nm. ACE inhibitory activity was calculated according to the following equation:


$$\mathrm{ACE}\;\mathrm{inhibitory activity}=\frac{A_{\mathrm{control}}-A_{\mathrm{sample}}}{A_{\mathrm{sample}}}\times 100\%$$

where, $A_{\mathrm{sample}}$ and $A_{\mathrm{control}}$ were the absorbance of the sample and control, respectively.

The IC_50_ value was determined by non-linear regression analysis using GraphPad Prism Version 6 software (GraphPad Software Inc., La Jolla, CA, United States), which calculated the peptide concentration of hydrolysate needed to achieve a 50% ACE inhibitory activity.

### 2.7 Inhibition kinetics of selected peptides

The ACE inhibition kinetics of the chosen peptides were determined using the method of previous research (20), using Lineweaver–Burk plots. The measurement of ACE activity was conducted at different concentrations of peptides, and HHL concentrations ranging from 2 to 5 mM were included. The Michaelis–Menten kinetic equation was used to compute the Michaelis–Menten constant (Km) and maximum reaction rate (Vmax) using data obtained from the Lineweaver–Burk plots.

### 2.8 Protective function on HUVECs of ACE inhibitory peptides

#### 2.8.1 Cell culture

The HUVECs were cultured in an incubator with DMEM culture fluid solution containing 10% fetal bovine serum, 1% bis-resistant (cyphenicillin mixture) at 37°C, and 5% CO_2_. Adherent cells were observed under a microscope, and the cell survival rate was above 95%.

#### 2.8.2 Cell viability assessment using MTT assay

The cell viability was determined according to the study by Suo et al. (21). The HUVECs (5 × 10^3^ cell/mL) were seeded into 96-well plates and cultured overnight at 37°C. Then, peptide or captopril (10 mg) was dissolved in DMEM (10 mL) and then was diluted to 50, 100, and 200 μg/mL, respectively. The diluted solution was added to the HUVECs and cultured for 48 h. The cells were added to 20 μL of MTT solution (5 mg/mL) and incubated for 4 h. Finally, DMSO (150 μL) was added to the mixture, and the absorbance was determined at 490 nm according to the study by Wang et al. (22).

#### 2.8.3 Evaluation of NO and ET-1 production

The NO and ET-1 contents of HUVECs were determined using an NO assay kit and an ET-1 assay kit, respectively.

### 2.9 Statistical analysis

The data were acquired by averaging three parallel experiments and are presented as means ± standard deviation (SD). To determine the significant distinctions between the samples, an analysis of variance (ANOVA) was conducted. The significance of the disparities in data was determined using Duncan’s test at a 95% confidence interval utilizing SPSS software (version 22.0).

## 3 Results and discussion

### 3.1 The ACE inhibitory activity of EBCHs

**Figure 1:**
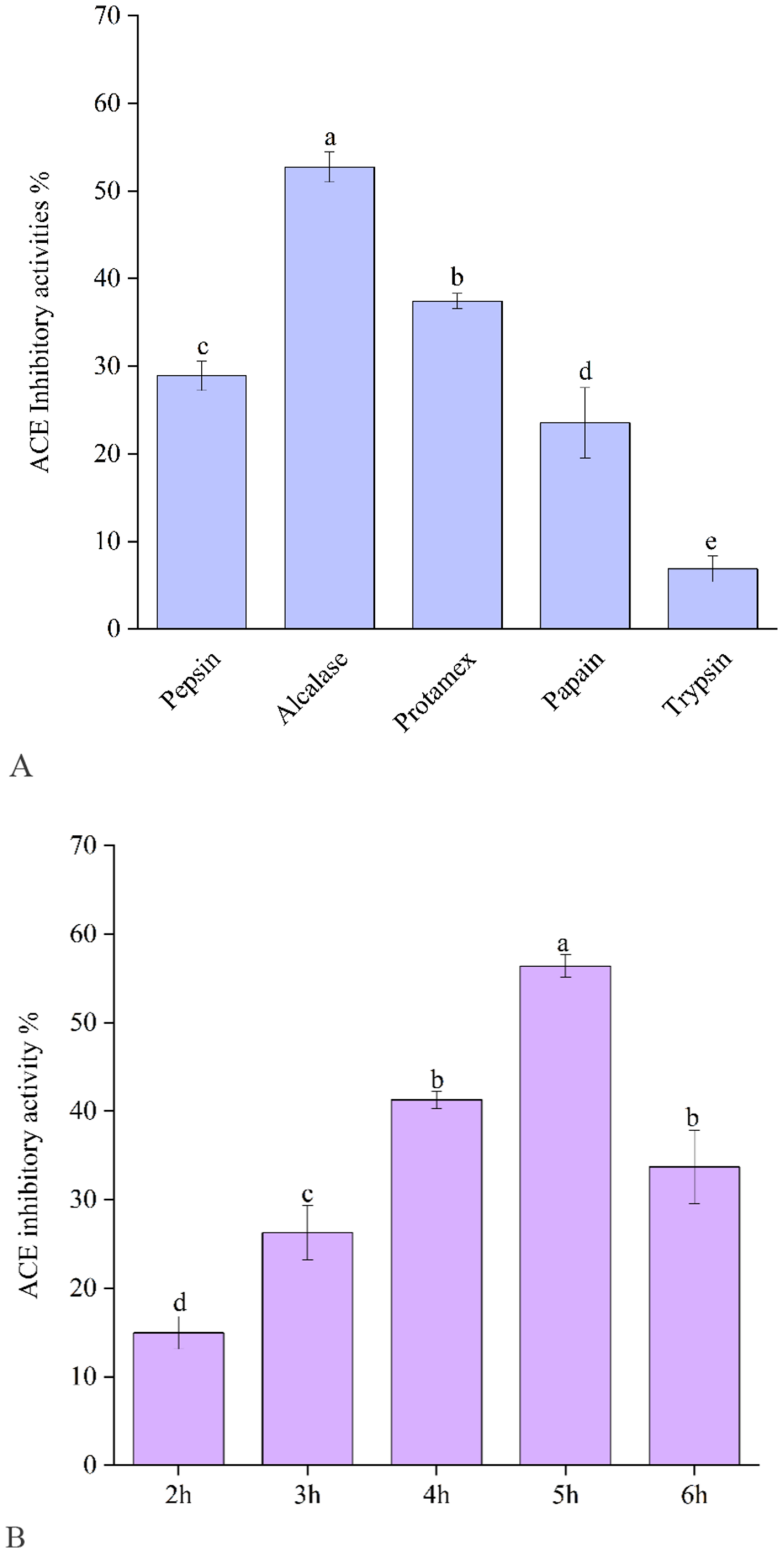
Angiotensin-I-converting enzyme (ACE) inhibitory activity of EBCHs obtained by trypsin, Alcalase, Protamex, papain, and trypsin, respectively **(A)**. ACE inhibitory activity of EBCHs obtained by Alcalase at different hydrolysis times **(B)**. ACE inhibitory activities of EBCHs were measured at 10 mg/mL. Different letters indicate statistical differences (*p* < 0.05).

According to the data shown in Figure 1A, Alcalase-produced EBCHs exhibited greater effectiveness in releasing peptides with ACE inhibitory effects (52.75 ± 1.75%), Protamex (37.43.75 ± 0.86%), pepsin (28.90 ± 1.68%), and papain (23.52 ± 4.02%) following behind. In contrast, EBCHs derived from trypsin (6.86 ± 1.46%) exhibited lower effectiveness due to cleavage taking place at varying locations in each protease. For instance, Alcalase-derived Alaska pollack (*Theragra chalcogramma*) skin hydrolysates showed the strongest ACE inhibitory effects compared with the other four proteases (23), and the pepsin hydrolysate from Pacific cod skin gelatin (1 mg/mL) showed the strongest ACE inhibitory effect of approximately 91% (24). Therefore, a suitable combination of food proteins and enzymes may generate protein hydrolysates with higher ACE inhibitory capacities. Subsequently, the impact of Alcalase’s duration of hydrolysis on ACE inhibition was examined. There were significant differences in the ACE inhibitory rate of the EBCHs at different times (*p* < 0.05). The ACE inhibitory rate of EBCHs (10 mg/mL for each sample) was the highest (50.58%) at 5 h. In comparison, it decreased to 36.12% as the hydrolysis time extended to 6 h (Figure 1B) due to the released ACE inhibitory peptides, which was over-hydrolyzed.

### 3.2 Separation of ACE inhibitory peptide from EBCHs

As shown in Supplementary Table S1, 93.57% of EBCHs-Alcalase has molecular weights (MWs) less than 3 kDa, of which 57.02% of EBCHs-Alcalase are less than 1 kDa. Therefore, EBCHs-Alcalase was separated into three fractions [F1 (>3 kDa), F2 (1–3 kDa), and F3 (<1 kDa)] through ultrafiltration membranes. The ACE inhibitory effect of F1, F2, and F3 was 3.08 ± 0.66%, 79.33 ± 0.17%, and 61.33 ± 1.89%, respectively, at 5 mg/mL. The F2 exhibited the greatest ACE inhibitory activity compared to the hydrolysates, aligning with previous findings that higher ACE inhibitory activity is associated with lower molecular weight (25). Nevertheless, the ACE inhibitory efficacy of F3 decreased slightly to 61.33 ± 1.89%, possibly attributable to the generation of free amino acids. Therefore, F2 was chosen to screen the potent peptides (Table 1).

**Table 1:** ACE inhibitory activity of the fraction separated by ultrafiltration (5 mg/mL).

Fraction	ACE inhibitory activity (%)
EBCHs-Alcalase	25.54 ± 0.11
MW >3 kDa	3.08 ± 0.66
1 kDa < MW < 3 kDa	79.33 ± 0.17
MW < 1 kDa	61.33 ± 1.89

### 3.3 Identification of peptides from F2 by peptidomics

**Figure 2:**
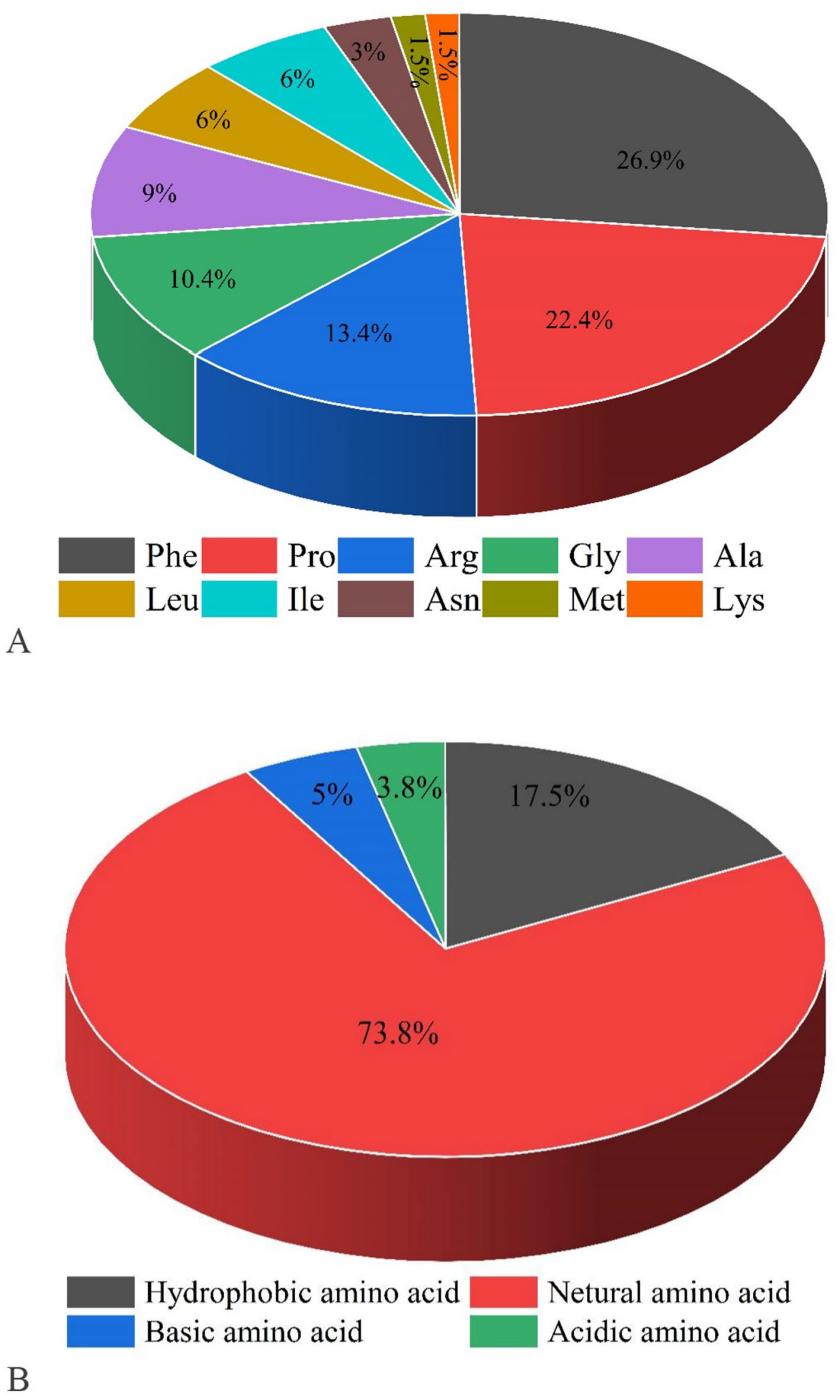
Proportions of C-terminal amino acids **(A)** and N-terminal amino acids for screening peptides **(B)**.

**Figure 3:**
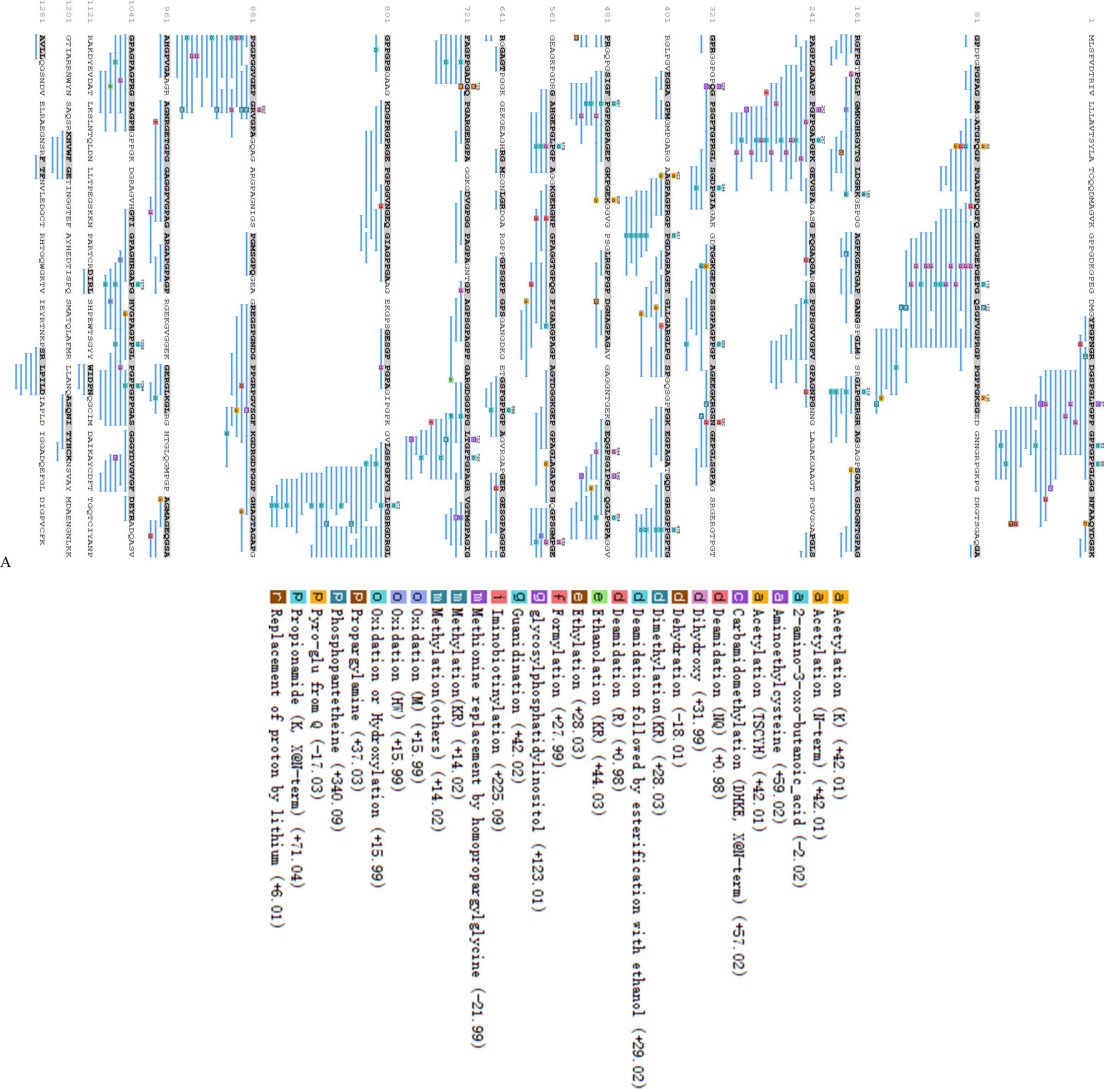

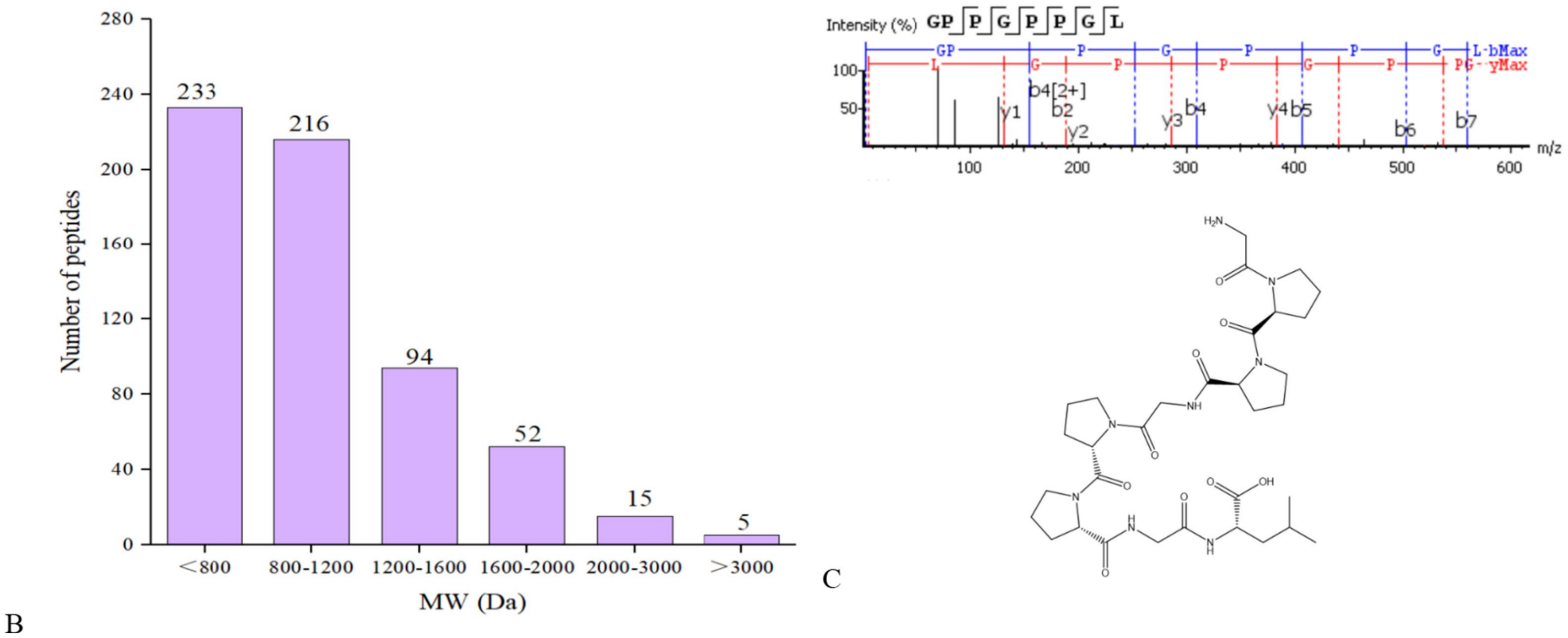
Peptide mapping of A0A090AX23_ANGJA that released GPPGPPGL **(A)**; number of peptides at different MWs identified from F2 **(B)**; MS/MS spectrum of GPPGPPGL and the chemical structure of GPPGPPGL **(C)**.

Following the elimination of duplicates, a total of 615 peptides originating from type I collagen in F2 were successfully identified. Figure 2B displays the distribution of molecular weights (MWs) for a total of 615 peptides, ranging from 243.12 to 4256.96 Da. Among these, 96.75% of peptides have MWs less than 2 kDa, indicating that their lower MWs may contribute to their potent ACE inhibitory properties. The representative peptide map, MS/MS map, and chemical structure of peptide GPPGPPGL released from A0A090AX23_ANGJA (61–68) are depicted in Figures 3A,C, respectively.

### 3.4 Screening of ACE inhibitory peptides

The remaining 615 peptides were screened using the Peptide Ranker program based on their structural patterns, and 67 peptides with scores of >0.8 (i.e., potentially bioactive) were obtained (Figure 3B). In a previous study, it was found that peptides containing hydrophobic and aromatic amino acids at the C-terminus exhibited a remarkably strong inhibitory effect on ACE, as stated in the previous publication (26). There are 10 kinds of C-terminal residues for 67 peptides, among which Phe and Pro were dominant in amount and accounted for 49.3% of peptides (26.9 and 22.4%, respectively). In addition, these peptides contained other hydrophobic amino acids such as Ala (9%), Leu (6%), Ile (6%), and Met (1.5%) (Figure 2A). The presence of these amino acids can contribute to enhancing the effectiveness of ACE inhibitory peptides. Furthermore, the size and electronic characteristics of amino acids play a role in the bioactivity of peptides (27). Hence, the presence of a benzene ring in Phe and the electronic properties in Arg can also enhance the size and promote the stability of the interaction between ACE and peptide, consequently leading to a significant increase in ACE inhibitory activity. Out of the total 67 peptides, 14 peptides (17.5%) have hydrophobic amino acid at the N-terminal, while 59 peptides (73.8%) have neutral amino acid at the N-terminal. In addition, 8.8% of the peptides have positively or negatively charged amino acid at the N-terminal, as shown in Figure 2B. Afterward, the initial peptides (67) underwent screening with pepsite2 (PepSite),^1^ resulting in the identification of 23 peptides exhibiting potential ACE inhibitory activity, with a significance level of *p* < 0.001 (Table 2). Moreover, peptides with reduced ACE binding effects showed decreased Vina scores, which were indicative of enhanced ACE inhibitory capacities. After preliminary selection, a total of 23 peptides were chosen based on their higher peptide ranker value and lower *p*-value, as indicated in Supplementary Table S2. Following that, a total of seven peptides (PMGPR, GPMGPR, GPAGPR, GPPGPPGL, GGPGPSGPR, GPIGPPGPR, and GPSGAPGPR) were synthesized, having both lower *p*-values and Vina scores as indicated in Table 2. The ACE inhibition activities (IC_50_) of each peptide were measured *in vitro*. The results given in Table 2 showed that the IC_50_ values for each synthetic peptide ranged from 535.84 to 3663.82 μM, while they were less than the captopril (IC_50_ = 7.5 nM), data are not shown in Table 2. Chen et al. (20) reported the potential ACE inhibitory peptides with IC_50_ values ranging from 41.06 to 6533.32 μM, while Panyayai et al. (28) reported that the peptides with IC_50_ values ranging from 0.32 to 1,000 μM had the potential to reduce blood pressure. Among the seven peptides, GPPGPPGL was the most potent ACE inhibitory peptide with IC_50_ values of 535.84 μM.

**Table 2:** Physiochemical characteristics, Vina score, IC_50_, and interactions of peptides virtually screened from EBCHs.

Number	Sequence	Mass (Da)	Size	Vina score (kCal/moL)	IC_50_ (μM)	Hydrogen bond	Hydrophobic interaction	Electrostatic	Other
1	PMGPR	556.2791	5	−9.0	2642.51	Tyr523 (2.52 Å), Ala356 (2.41 Å), Ser355 (2.96 Å), Ala354 (3.27 Å, 3.51 Å), and Glu384 (3.54 Å)	His387, Val518, Phe527, and Val380	Glu411	His353 Zn701
2	GPMGPR	613.3006	6	−8.8	3663.82	Ala356 (2.69 Å), Ser355 (2.59 Å), Tyr523 (2.28 Å), Ala354 (3.28 Å, 3.64 Å), Glu384 (3.37 Å), Glu411(2.19 Å), and Pro407(2.54 Å)	Phe457, Phe527, His383, Tyr523, Val380, His353, Phe512, and Val518	His383, Glu411, and His410	Zn701
3	GPAGPR	553.2972	6	−9.0	2251.88	Asp415 (2.38 Å), His383 (2.95 Å), Tyr523 (2.79 Å, 2.24 Å), Ala354 (1.91 Å), Arg522 (6.24 Å), Ala356 (2.19 Å), and His387 (2.92 Å)	Phe457 and Val518	His387, Glu411, and His410	Zn701
4	GPPGPPGL	690.3701	8	−8.4	535.84	Glu411(3.19 Å), Ala356 (2.72 Å), Arg522 (6.02 Å), His353 (2.53 Å), His513 (2.52 Å, 2.81 Å), Lys511(2.28 Å), and Gln281(2.22 Å)	Pro407, His410, His387, Tyr523, and Phe457	Glu411, His383, and Glu384	Phe391, Glu403, Trp357, Tyr520, Val518, Phe512, Gly404, Ser355, Zn701, Ala354, Val380, and Phe527
5	GGPGPSGPR	780.3878	9	−8.4	971.31	Ala356 (1.97 Å), Ser355 (2.49 Å), Arg522 (1.93 Å), Ala354 (2.35 Å), His353 (1.81 Å), His513 (2.06 Å), Tyr520 (3.30 Å), Gln281 (5.10 Å), and Arg402 (2.90 Å)	Val518, His383, Tyr523, and Phe527	–	Zn701
6	GPIGPPGPR	846.4711	9	−6.4	1702.42	Tyr523 (2.63 Å and 2.60 Å), Ala354(3.07 Å), Asp415(4.56 Å), Gln281 (2.39 Å), and Arg522 (2.34 Å)	Phe512, Val380, His353, Ala354, His410, His387, and Phe391	Asp415	Zn701
7	GPSGAPGPR	794.4034	9	−8.5	1261.36	Gln281 (2.84 Å and 2.45 Å), Ala354(3.35 Å and 3.54 Å), and Asp415 (3.20 Å)	Pro407, His410, Val518, Phe512, His387, Ala356, Ala354, His353, Val380, and His383	Asp415	Zn701

### 3.5 Molecular docking of GPPGPPGL with ACE

**Figure 4:**
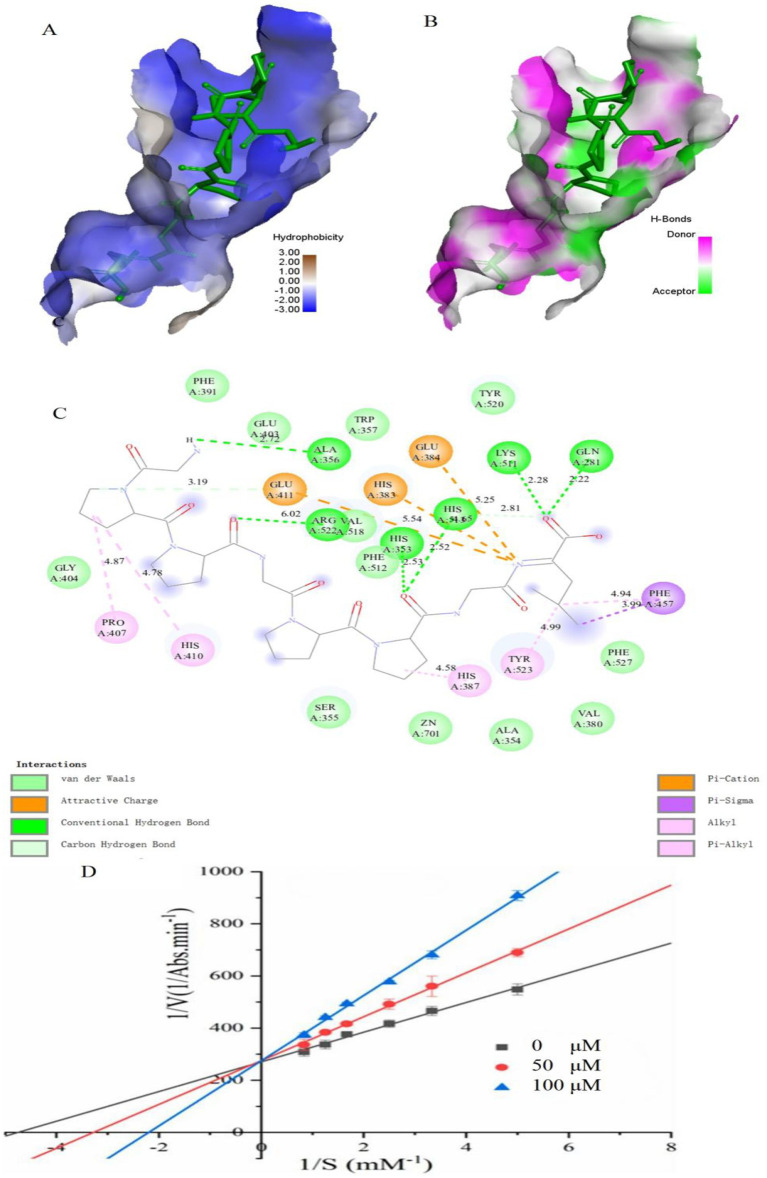
Molecular docking results for GPPGPPGL with ACE (PDB:1O8A) and inhibition mode. **(A)** 3D hydrophobic interaction of GPPGPPGL at the active site. **(B)** 3D hydrogen interaction of GPPGPPGL at the active site. **(C)** 2D interaction details for GPPGPPGL. **(D)** Lineweaver–Burk plots of the ACE inhibition of GPPGPPGL at three concentrations.

Molecular docking is to further clarify the interaction and potential binding sites between amino sequence with the crystal structure of ACE (entry code: 1O8A) was acquired from the Protein Data Bank. The molecular docking interaction between ACE and the peptides of GPPGPPGL was simulated using AutoDock 4.2 software. GPPGPPGL can form a stable docking structure with ACE, which has a binding energy value of −8.4 kcal/mol (Figure 4). GPPGPPGL and ACE residues are mainly connected by hydrogen bonds, hydrophobic interactions, polarity, van der Waals force, and static power connections (19). The interaction of hydrogen bonds plays a crucial part in the formation of stable docking complexes involving enzyme catalytic reactions (29). ACE possesses three primary sites for activity, which consist of S1 (Ala354, Glu384, and Tyr523), S2 (Gln281, His353, Lys511, His513, and Tyr 520), and S′1 (Glu 162) active site pockets2 (30).

The ACE conformation could be influenced by the following four types of interactions in relation to GPPGPPGL: hydrogen bonds (including conventional hydrogen bond and carbon–hydrogen bond), hydrophobic interactions (such as Alkyl, Pi-Sigma, and Pi-Alkyl), electrostatic interactions (including attractive charges and Pi-Cation), and van der Waals interactions. GPPGPPGL established a total of eight hydrogen bonds with Glu411, Ala356, Arg522, His353, His513, Lys511, and Gln281 within ACE at a distance of 3.19 Å, 2.72 Å, 6.02 Å, 2.53 Å, 2.52 Å/2.81 Å, 2.28 Å, and 2.22 Å, respectively (as shown in Table 2). Among them, Gln281, His353, and His513 belonged to S2 pockets. Furthermore, the Pro in GPPGPPGL can form alkyl bonds with Pro 407, His 410, and His 387 of ACE. In addition, the Leu residue in GPPGPPGL can form alkyl bonds with Tyr 523 (located in the S1 pocket) and engage in pi-alkyl/pi-sigma interactions with Phe 457. These findings indicated that the hydrophobic interaction significantly influences the ACE inhibitory activity. The appealing positive charge and Pi-cation created by Leu in the peptide, along with Glu411, Glu384, and His383, can also influence the ACE inhibitory activity. In addition, ACE contains residues such as Phe391, Glu403, Trp357, Tyr520, Val518, Phe512, Gly404, Ser355, Zn701, Ala354, Val380, and Phe527, which can interact with GPPGPPGL through Van der Waals forces. The findings indicated that GPPGPPGL can interact efficiently with residues at the activity sites, which could potentially be crucial for exhibiting ACE inhibitory activity. The molecular docking results support the notion that GPPGPPGL exhibits greater ACE inhibitory activity due to its increased number of hydrogen bonds, attractive charge, and Pi-cation interaction. The Pi interaction’s significance in peptide binding with ACE was in line with previous research findings (31).

### 3.6 Inhibition mode of ACE inhibitory peptides

Previous research has shown that peptides derived from various origins have exhibited several mechanisms of ACE inhibition, such as competitive, non-competitive, and mixed inhibition (32). Figure 4D shows the Lineweaver–Burk plots obtained for GPPGPPGL. With the increase in the levels of GPPGPPGL (0–100 μM), Km showed an increase, while Vmax stayed constant, indicating competitive inhibition (33). We hypothesized that GPPGPPGL engaged with the ACE active sites, hindering substrate binding and consequently diminishing the enzyme’s catalytic function.

### 3.7 Protective function on HUVECs of GPPGPPGL

**Figure 5:**
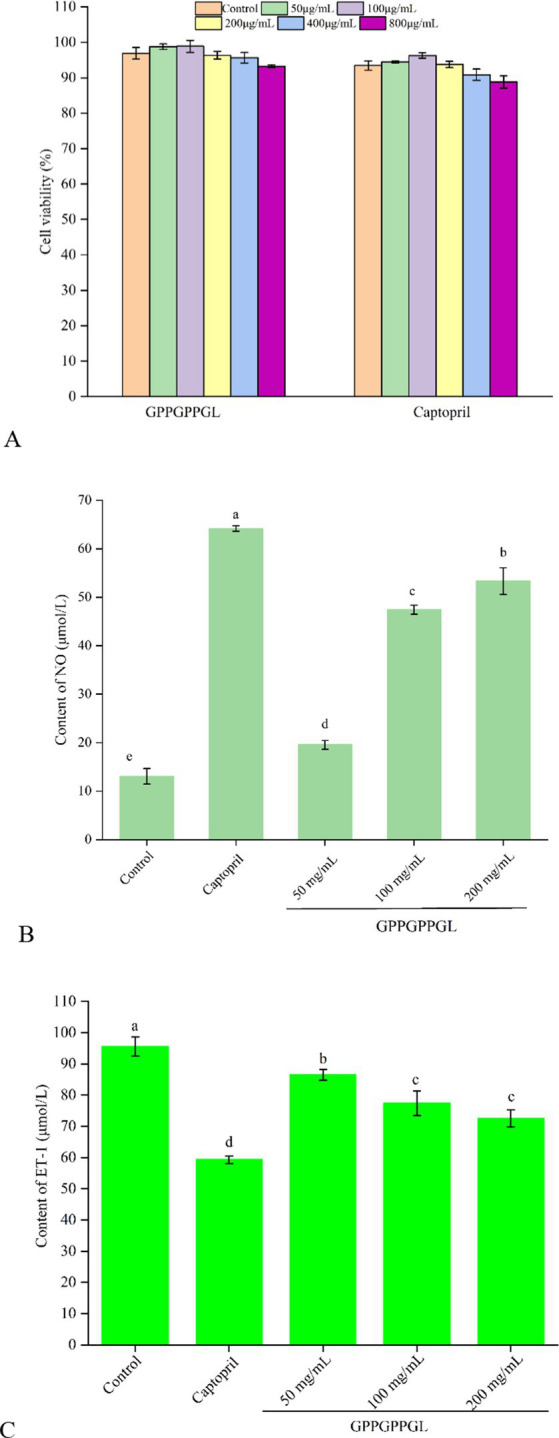
Protective function on HUVECs of GPPGPPGL. **(A)** The cell viability of HUVECs treated with GPPGPPGL for 48 h. **(B)** The production of nitric oxide (NO) in HUVECs treated with GPPGPPGL for 48 h. **(C)** The endothelin-1 (ET-1) secretion of HUVECs treated with GPPGPPGL for 48 h.

As shown in Figure 5A, there was no significant difference in the viability of HUVECs, which were treated at different concentrations (50, 100, 200, 400, and 800 μg/mL) of GPPGPPGL and captopril for 48 h. HUVECs were the surface cell in vascular, which can produce a variety of substances related to the balance of blood pressure, such as vascular diastolic factor nitric oxide (NO) and vascular shrinkage factor ET-1. Peptides have significant proliferation or inhibitory effects on the normal growth of HUVECs, which will affect the integrity of cell organ structure (34). As shown in Supplementary Figure S1, HUVECs were cultured at different concentrations (0, 50, 100, and 200 μg/mL) of GPPGPPGL for 48 h, and there was no significant difference in cell morphology under the microscope. The shape of HUVECs is spindle-like, and the boundaries between cells are clear. GPPGPPGL has no significant effect on cell growth as concentration increases. As shown in Figure 5B, there was significant difference in NO contents between cells treated with GPPGPPGL for 48 h at different concentrations (50, 100, and 200 μg/mL). NO antagonizes the effects of angiotensin II on vascular tone, cell growth, and renal sodium excretion and also downregulates the synthesis of ACE and angiotensin II type I receptors (35). Huang et al. (36) reported that the peptides YH identified from yeast hydrolysates can promote the release of NO. Therefore, GPPGPPGL can effectively promote NO release and has the potential to lower blood pressure.

As shown in Figure 5C, the ET-1 contents in HUVECs significantly decreased after 48-h treatment at different concentrations (50, 100, and 200 μg/mL) of GPPGPPGL (*p* < 0.05). Within a certain range, the ET-1 content showed a higher dose dependence with the GPPGPPGL concentration. However, the effect of the GPPGPPGL was lower than the effect of captopril. Endothelin (ET) includes endothelin-1 (ET-1), endothelin-2 (ET-2), and endothelin-3 (ET-3), where ET-1 is a vascular shrinkage that mainly affects blood vessel shrinkage (37). It can trigger vascular dysfunction related to cardiovascular diseases such as hypertension and atherosclerosis (35). Zhao et al. (38) reported that Antarctic krill inhibitory peptides of KAP1, KAP3, KAP6, and KAP7 in concentrations of 50, 100, and 200 μmol/L have an inhibitory effect on the secretion of ET-1.

## 4 Conclusion

In summary, ECBHs with the highest ACE inhibitory effect (IC_50_ = 10.66 mg/mL) were obtained by Alcalase for 4 h. The identification of 615 peptides was achieved by ultrafiltration membranes followed by peptidomics. Subsequently, seven ACE inhibitory peptides (PMGPR, GPMGPR, GPAGPR, GPPGPPGL, GGPGPSGPR, GPIGPPGPR, and GPSGAPGPR) were virtually screened, and the most potent was GPPGPPGL with IC_50_ values of 535.84 μM. Furthermore, GPPGPPGL displayed a competitive inhibitory effect on ACE primarily as a result of the formation of multiple hydrogen bonds and hydrophobic interactions with the S1 and S2 pockets. The strong inhibitory effect of peptides against ACE is mainly attributed to the hydrophobic residues located at the N-terminus, which is of greater significance. Likewise, the peptides’ C-terminal amino acids have a notable impact on their capacity to hinder ACE. The increased ACE inhibitory activity of GPPGPPGL was demonstrated through molecular docking, which was attributed to its greater number of hydrogen bonds, attractive charge, and Pi-cation interactions. GPPGPPGL significantly (*p* < 0.01) promoted nitric oxide secretion and reduced endothelin-1 production. A further study should be carried out to examine the bioavailability and *in vivo* activity of peptide GPPGPPGL identified from EBCHs.

## Data Availability

The original contributions presented in the study are included in the article/Supplementary material, further inquiries can be directed to the corresponding authors.

## Footnotes

^1^ http://pepsite2.russelllab.org/
