# Supplementary material for: Angiotensin-I-converting enzyme inhibitory peptides from eel (Anguilla japonica) bone collagen: preparation, identification, molecular docking, and protective function on HUVECs
Source: Front Nutr. 2024 Dec 5;11:1462656. doi: 10.3389/fnut.2024.1462656 (PMC11655196; doi:10.3389/fnut.2024.1462656)
Supplement: Supplementary file 1 [file Table_1.DOCX]

Table S1 The MWs distribution profiles of EBCHs-Alcalase

| Number | MWs (kDa) | Percentage (%) |
| --- | --- | --- |
| 1 | ˃5 | 1.71 |
| 2 | 3-5 | 4.72 |
| 3 | 1-3 | 36.55 |
| 4 | ˂1 | 57.02 |

Table S2 The Peptides Virtually Screened from EBCHs

| Amino acids sequence | MW Da | Length | Peptide ranker | p-value | Vina score |
| --- | --- | --- | --- | --- | --- |
| PMGPR | 556.2791 | 5 | 0.872946 | 0.0000002871 | -9.0 |
| GPMGPR | 613.3006 | 6 | 0.891703 | 0.000003245 | -8.8 |
| GPAGPR | 553.2972 | 6 | 0.801968 | 0.000003568 | -9.0 |
| GPPGPPGL | 690.3701 | 8 | 0.956284 | 0.0000050 | -8.4 |
| GGPGPSGPR | 780.3878 | 9 | 0.804042 | 0.00001001 | -8.4 |
| GPIGPPGPR | 846.4711 | 9 | 0.897042 | 0.00001029 | -6.4 |
| GPAGPAGPR | 778.4086 | 9 | 0.860728 | 0.00001602 | -7.6 |
| GPSGAPGPR | 794.4034 | 9 | 0.848616 | 0.00002637 | -8.5 |
| GFPGPK | 601.3224 | 6 | 0.915372 | 0.00003174 | -9.3 |
| FPGAP | 487.243 | 5 | 0.948409 | 0.00004384 | -8.6 |
| GFPGLP | 586.3115 | 6 | 0.96333 | 0.00006682 | -8.6 |
| GPPGRP | 579.3129 | 6 | 0.907099 | 0.0000835 | -9.9 |
| FPGLP | 529.29 | 5 | 0.966222 | 0.00008714 | -8.9 |
| PPGPPGGGF | 781.3759 | 9 | 0.971048 | 0.00008876 | -8.1 |
| RGPPGPM | 710.3533 | 7 | 0.899236 | 0.0000933 | -6.0 |
| QGF | 350.159 | 3 | 0.940972 | 0.0001369 | -7.0 |
| GPPGFPGGA | 755.3602 | 9 | 0.943189 | 0.0001866 | -4.6 |
| GPAGF | 447.2118 | 5 | 0.937737 | 0.0002156 | -8.4 |
| WIDPN | 643.2966 | 5 | 0.95597 | 0.0002541 | -8.6 |
| GPSGPPGPS | 751.35 | 9 | 0.900525 | 0.0004637 | -8.0 |
| FGFI | 482.2529 | 4 | 0.980717 | 0.0004664 | -9.3 |
| HVWFG | 644.3071 | 5 | 0.901926 | 0.0006317 | -6.6 |
| AGFQGLP | 688.3544 | 7 | 0.845175 | 0.0008133 | -8.3 |
