# Supplementary material for: Angiotensin-I-converting enzyme inhibitory peptides from eel (Anguilla japonica) bone collagen: preparation, identification, molecular docking, and protective function on HUVECs
Source: Front Nutr. 2024 Dec 5;11:1462656. doi: 10.3389/fnut.2024.1462656 (PMC11655196; doi:10.3389/fnut.2024.1462656)
Supplement: Supplementary file 2 [file Image_1.pdf]

Figure S1

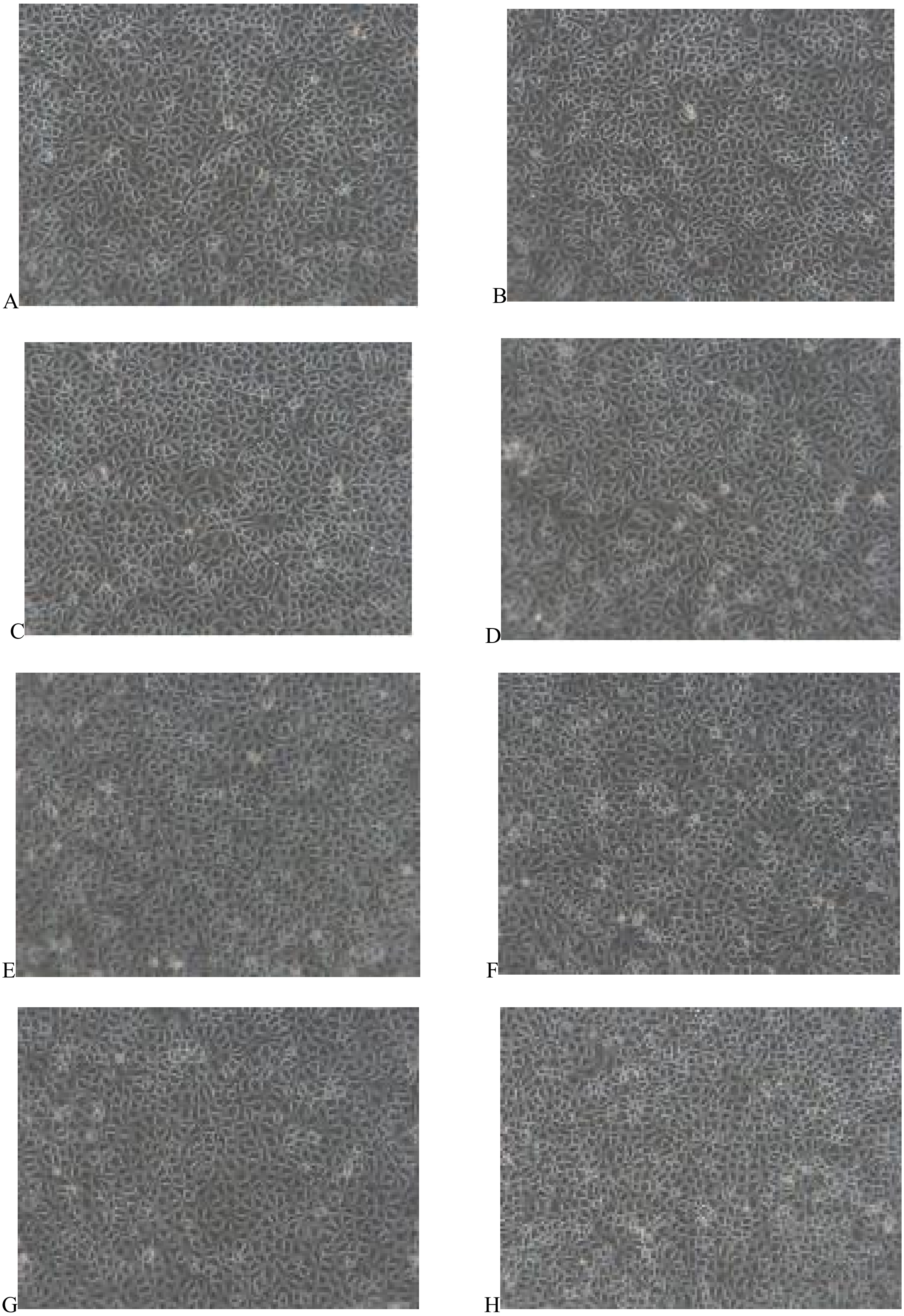

The morphology of HUVECs after GPPGPPGL treated. (A) GPPGPPGL: 0 μg/mL; (B) GPPGPPGL: 50 μg/mL; (C) GPPGPPGL: 100 μg/mL; (D) GPPGPPGL: 200 μg/mL; (E) captopril:

0 μg/mL; (F) captopril: 50 μg/mL; (G) captopril: 100 μg/mL; (H) captopril: 200 μg/mL.
