# Supplementary figures and images for: Angiotensin-I-converting enzyme inhibitory peptides from eel (Anguilla japonica) bone collagen: preparation, identification, molecular docking, and protective function on HUVECs
Source: Front Nutr. 2024 Dec 5;11:1462656. doi: 10.3389/fnut.2024.1462656 (PMC11655196; doi:10.3389/fnut.2024.1462656)

Figure S2

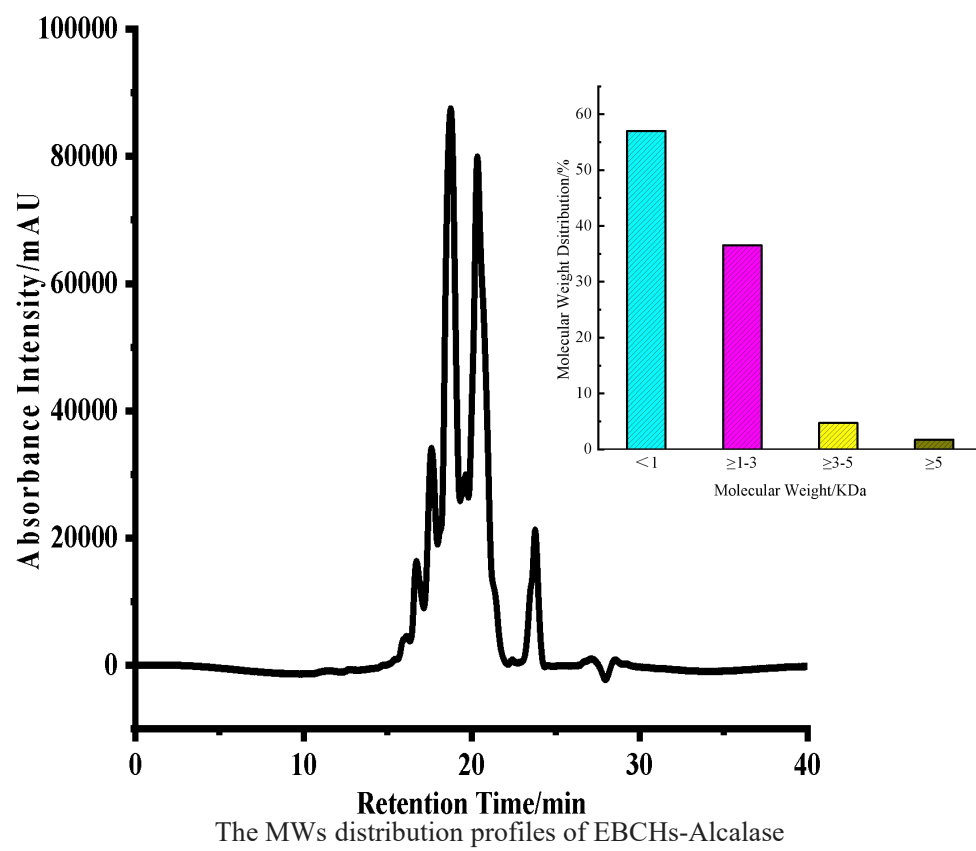

Supplement: Supplementary file 3 [file Image_2.pdf]
