# Supplementary material for: Angiotensin-I-converting enzyme inhibitory peptides from eel (Anguilla japonica) bone collagen: preparation, identification, molecular docking, and protective function on HUVECs
Source: Front Nutr. 2024 Dec 5;11:1462656. doi: 10.3389/fnut.2024.1462656 (PMC11655196; doi:10.3389/fnut.2024.1462656)
Supplement: Supplementary file 4 [file Image_3.pdf]

Figure S3: Molecular docking results for identified peptides with ACE (PDB:1O8A)

PMGPR

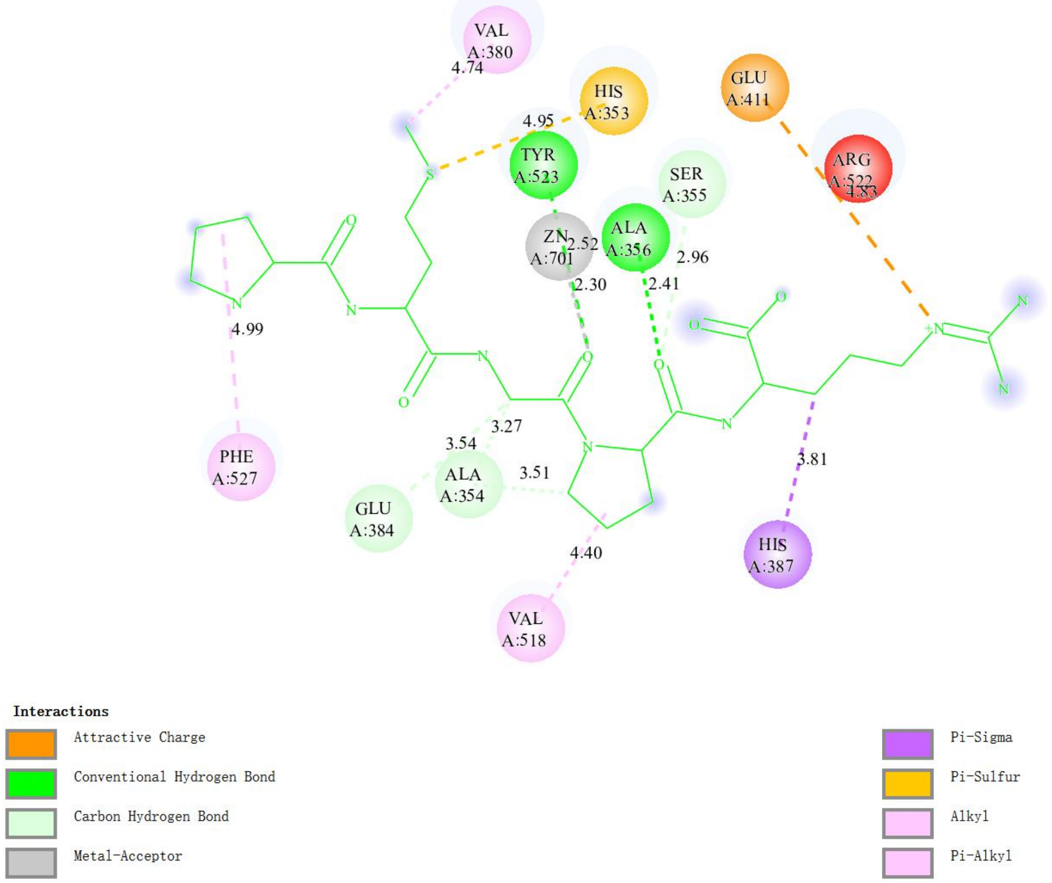

GPMGPR

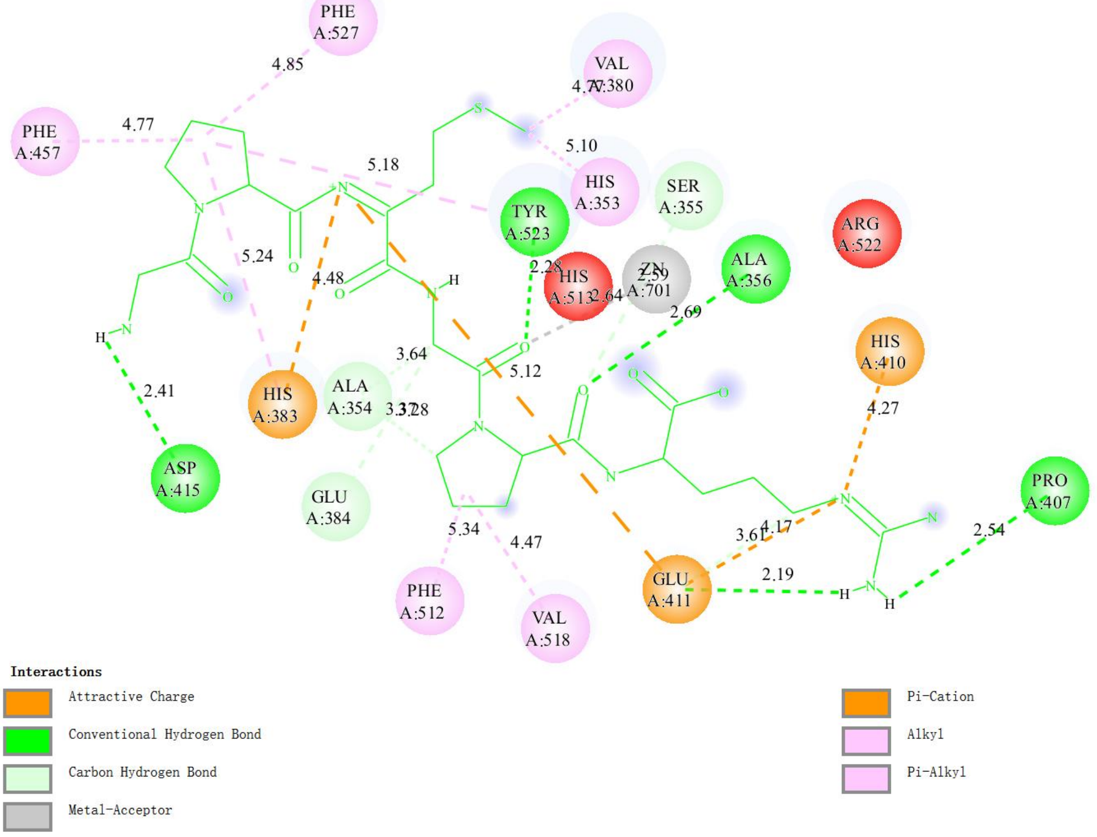

GPAGPR

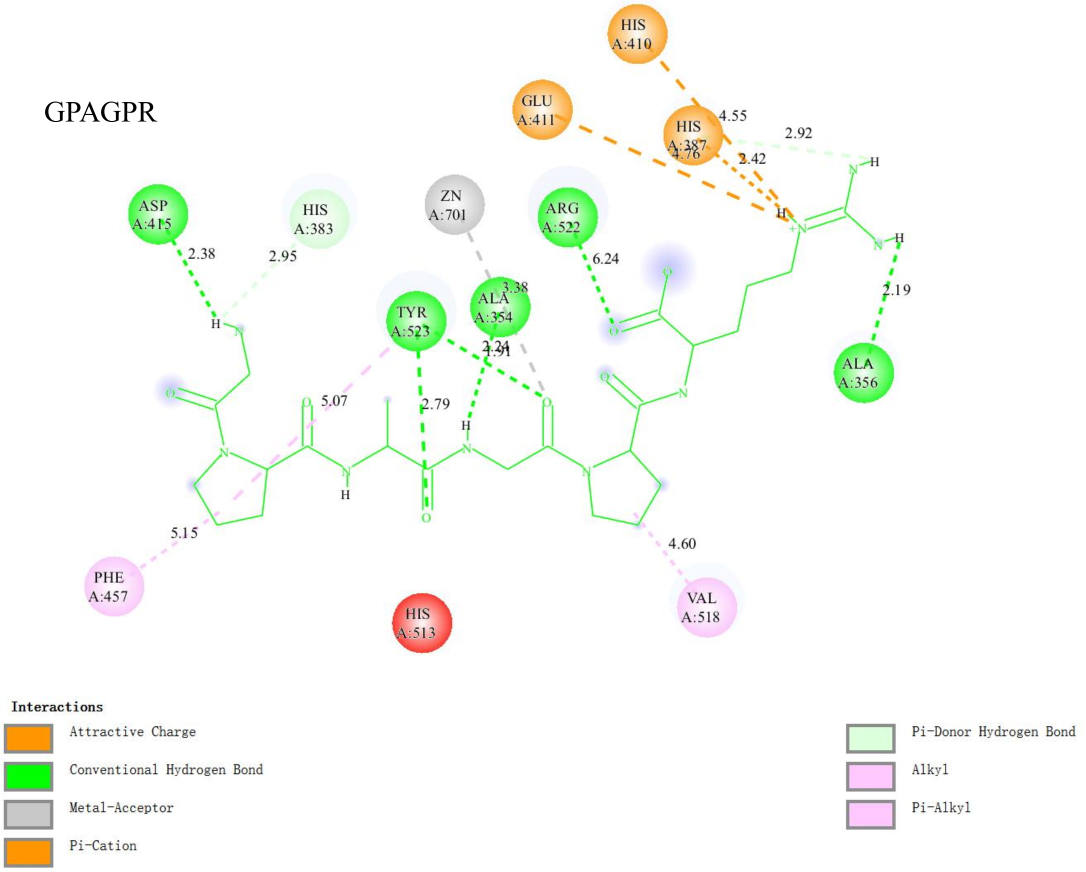

GPPGPPGL

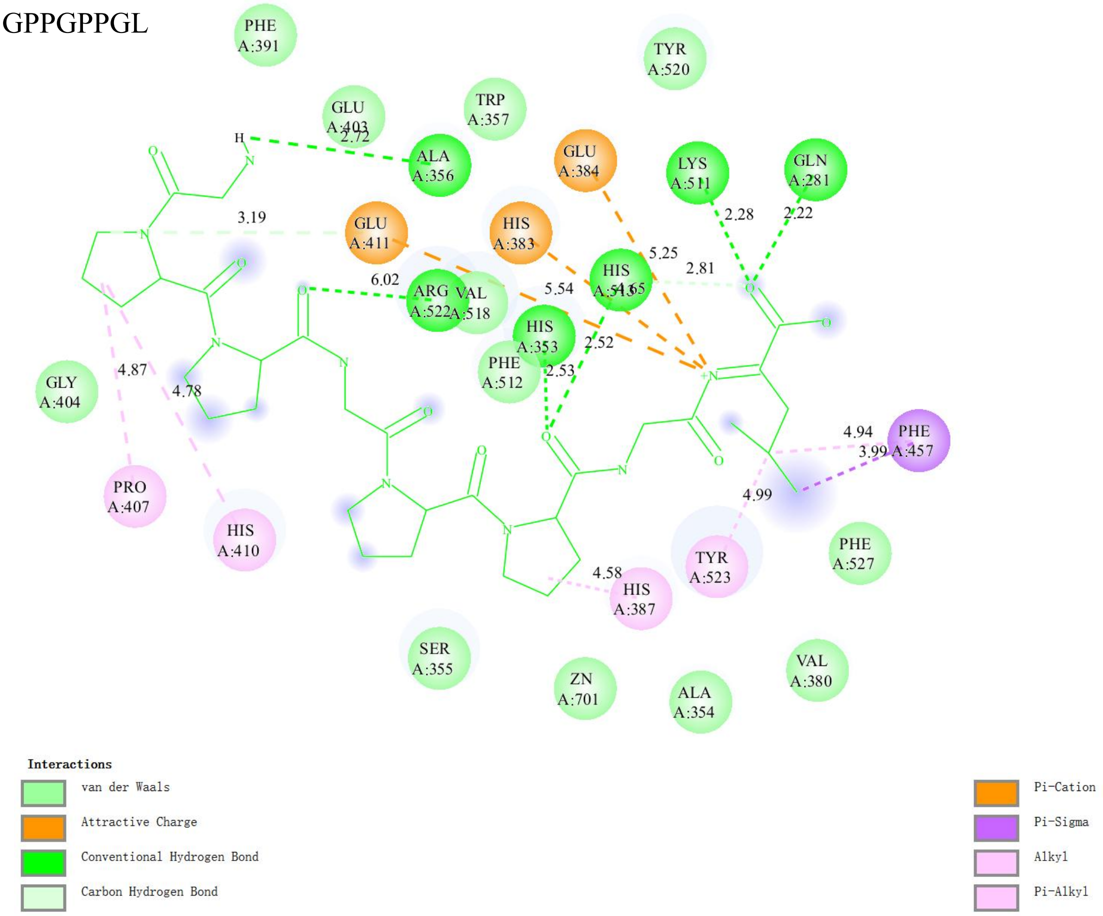

PHE A:391
TYR A:520
GLU A:403
TRP A:357
ALA A:356
GLU A:384
LYS A:511
GLN A:281
GLU A:411
HIS A:383
HIS A:513
ARG A:522
VAL A:518
HIS A:353
PHE A:512
GLY A:404
PRO A:407
HIS A:410
PHE A:457
PHE A:527
TYR A:523
HIS A:387
SER A:355
ZN A:701
ALA A:354
VAL A:380
2.72
3.19
6.02
5.54
5.25
2.28
2.22
2.81
4.65
2.52
2.53
4.87
4.78
4.94
3.99
4.99
4.58
Interactions
van der Waals
Attractive Charge
Conventional Hydrogen Bond
Carbon Hydrogen Bond
Pi-Cation
Pi-Sigma
Alkyl
Pi-Alkyl

GGPGPSGPR

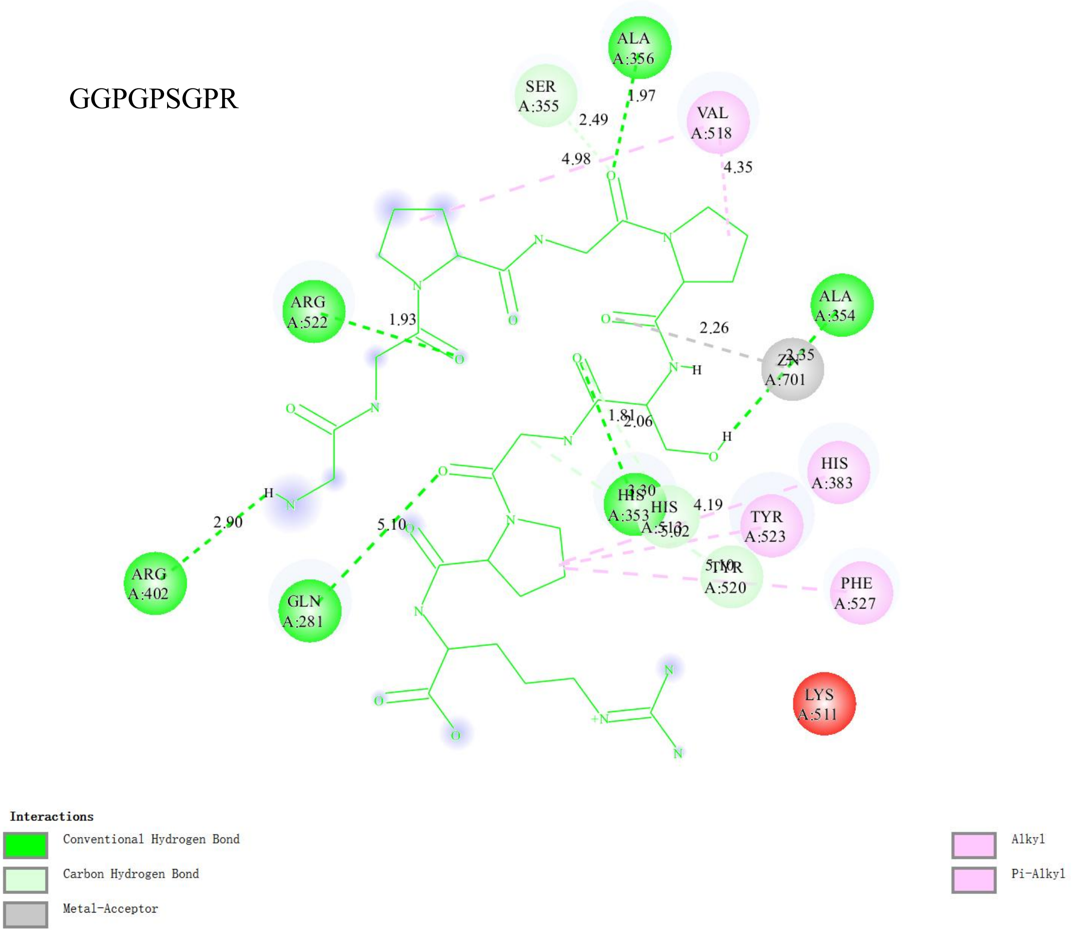
ALA A:356
SER A:355
VAL A:518
ARG A:522
ALA A:354
ZN A:701
HIS A:383
HIS A:353
TYR A:523
TYR A:520
PHE A:527
ARG A:402
GLN A:281
LYS A:511
1.97
2.49
4.98
4.35
1.93
2.26
2.35
1.81
2.06
4.19
2.90
5.10
Interactions
Conventional Hydrogen Bond
Carbon Hydrogen Bond
Metal-Acceptor
Alkyl
Pi-Alkyl

GPIGPPGPRR

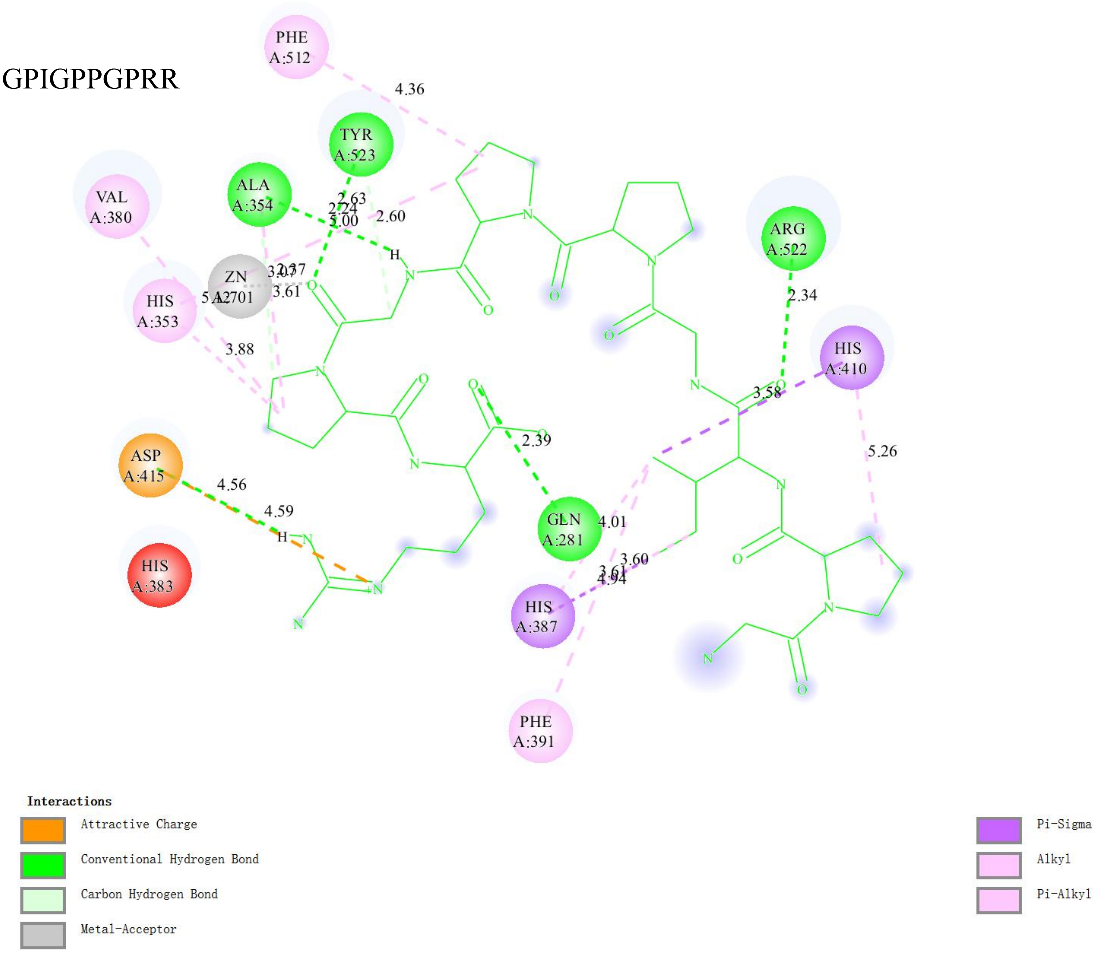
PHE A:512
TYR A:523
ALA A:354
VAL A:380
ZN A:2701
HIS A:353
ARG A:522
HIS A:410
ASP A:415
HIS A:383
GLN A:281
HIS A:387
PHE A:391
4.36
2.60
2.34
3.58
5.26
3.88
3.61
4.56
4.59
2.39
4.01
3.60
Interactions
Attractive Charge
Conventional Hydrogen Bond
Carbon Hydrogen Bond
Metal-Acceptor
Pi-Sigma
Alkyl
Pi-Alkyl

GPSGAPGPR

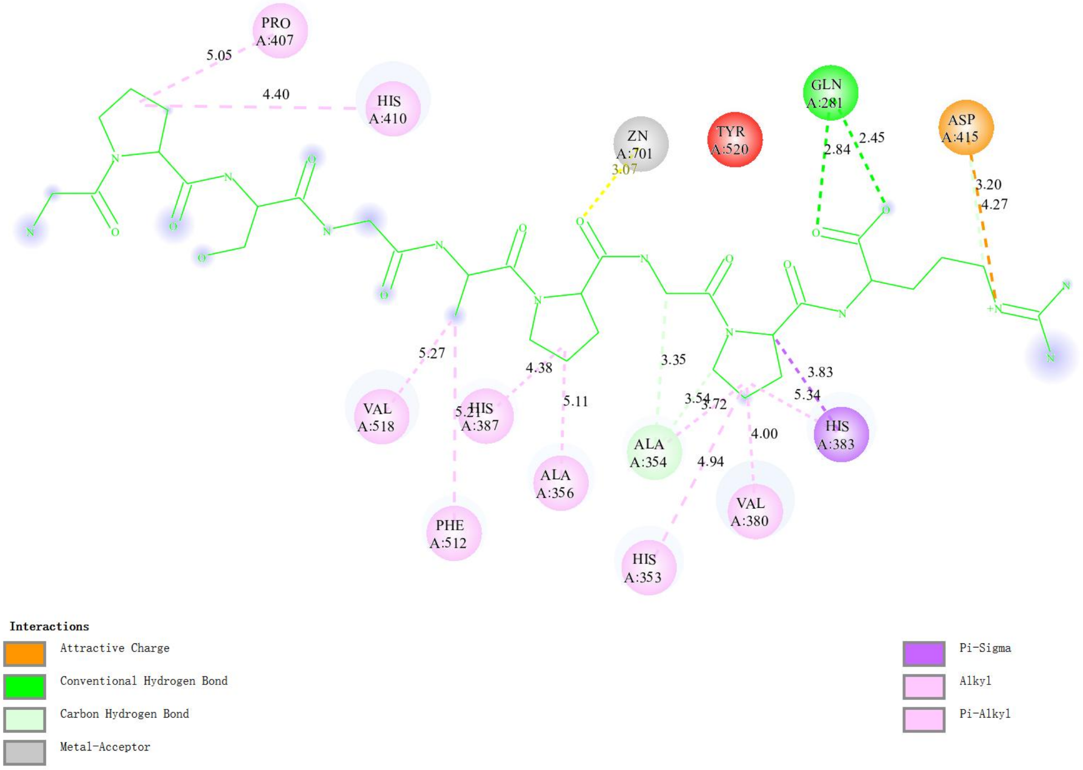
PRO A:407
HIS A:410
ZN A:701
TYR A:520
GLN A:281
ASP A:415
VAL A:518
HIS A:387
ALA A:356
PHE A:512
ALA A:354
HIS A:383
VAL A:380
HIS A:353
5.05
4.40
3.07
2.84
2.45
3.20
4.27
5.27
5.21
4.38
5.11
3.35
3.54
3.72
3.83
5.34
4.00
4.94
Interactions
Attractive Charge
Conventional Hydrogen Bond
Carbon Hydrogen Bond
Metal-Acceptor
Pi-Sigma
Alkyl
Pi-Alkyl
